# Green syntheses of silver nanoparticle decorated reduced graphene oxide using l-methionine as a reducing and stabilizing agent for enhanced catalytic hydrogenation of 4-nitrophenol and antibacterial activity†

**DOI:** 10.1039/c9ra08536j

**Published:** 2019-11-28

**Authors:** Neway Belachew, Desta Shumuye Meshesha, Keloth Basavaiah

**Affiliations:** Department of Chemistry, Debre Berhan University Debre Berhan Ethiopia neway.du@gmail.com neway@dbu.edu.et; Department of Chemistry, University of Gondar Gondar Ethiopia; Department of Inorganic & Analytical Chemistry, Andhra University Visakhapatnam-530003 Andhra Pradesh India

## Abstract

Herein, we have reported a facile and green synthesis approach of Ag NP decorated reduced graphene oxide (RGO) through an *in situ* self-assembly method in the presence of l-methionine (l-Met) as reducing and stabilizing agent. The electronic properties, crystal structure, and morphology of the as-synthesized RGO–Ag nanocomposite were investigated by UV-Visible (UV-Vis) spectroscopy, Fourier transform-infrared (FTIR), X-ray diffraction (XRD), field emission scanning electron microscopy (FESEM) and transmission electron microscopy (TEM) techniques. UV-Vis and FTIR show the effective reduction of GO and the formation of Ag NPs using l-Met. FESEM, TEM, and XRD analysis show the successful impregnation of Ag NPs into RGO with a 23 nm average crystallite size. The RGO–Ag nanocomposite with NaBH_4_ shows a fast-catalytic reduction of 4-nitrophenol (4-NP) to 4-aminophenol (4-AMP). The enhanced catalytic activity of RGO–Ag nanocomposites can be attributed to the synergistic effect of improved adsorption capacity and the absence of agglomeration of Ag nanoparticles. Moreover, RGO–Ag showed strong antibacterial activity against *B. subtilis* and *E. coli*.

## Abstract 2

Herein, we have reported a facile and green synthesis approach of Ag NP decorated reduced graphene oxide (RGO) through an *in situ* self-assembly method in the presence of l-methionine (l-Met) as reducing and stabilizing agent.

## Graphical abstract

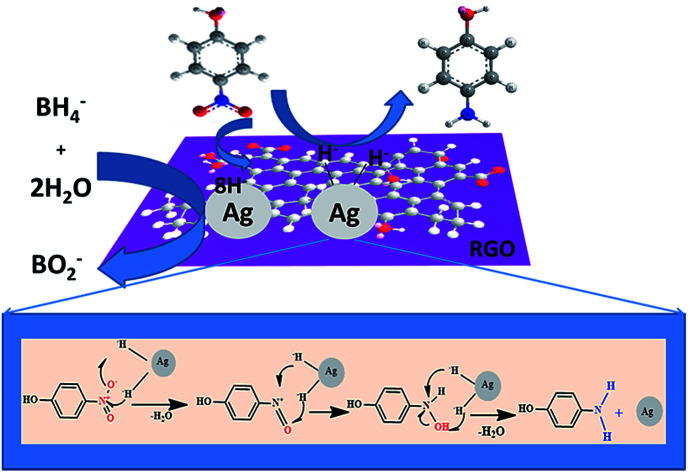

## 1. Introduction

During recent decades, the synthesis of metal nanoparticles (NPs) has received substantial attention for fundamental and applied research owing to their unique physical and chemical properties compared to bulk metals.^1,2^ They have a wide range of applications including drug delivery,^3^ sensing^4,5^ and catalysis.^6,7^ Among noble metal nanoparticles, Ag NPs have received much attention due to their stability, good conductivity, and high catalytic,^8^ antibiofouling,^9^ antibacterial,^10–12^ anticancer,^13^ anti-viral and antifungal activities.^1,14^ Furthermore, Ag is a relatively cheap metal catalyst as compared to Au. However, unprotected Ag NPs are vulnerable to irreversible agglomeration due to their high surface area to volume ratio, resulting in a remarkable reduction of their intriguing properties. In order to overcome these, there are numerous recent reports can be found in the preparation of protected Ag nanoparticles.^15^ Such as, synthesis of metal nanoparticles supported by polymers,^15^ surfactants,^16^ porous carbon materials,^17^ and graphene^18^ and shows enhanced efficiency for their respective applications. Among this graphene is reported to be promising nanoscale platform of new multifunctional composite materials.^19,20^ Graphene is a two-dimensional sp^2^-hybridized carbon material, due to its excellent charge transport mobility, large specific surface area, high electrocatalytic activity, and low cost currently attract the huge interest for the wide range of application.^21–23^

The graphene–Ag nanocomposites have been exploited for multifunctional applications, including catalysis,^24^ sensor,^25^ imaging,^26,27^ and energy storage.^28^ Hence, in order to explore the potential application, it is of prime importance to the synthesis of these nanocomposite materials through a facile, large-scale production with quality and environmentally friendly approaches. Numerous researches have been reported for the synthesis of RGO–Ag nanocomposites using different reducing agents such as hydrazine,^29^ NaBH_4_,^30^ amines,^31^ dimethylformamide,^32^ lactulose.^33,34^ However, nanocomposites prepared from such chemicals usually suffer from aggregation and poor aqueous dispersibility which limits the potential application of the nanocomposite material. Moreover, the chemicals are highly noxious in nature, which is harmful to both the environment and humans. Hence, it needs to search for an alternative environmentally friendly reagent.

Herein, we report a simultaneous reduction of GO and Ag^+^ using l-methionine (l-Met) in an *in situ* approach for the preparation of RGO–Ag nanocomposite. l-Met is an essential sulfur-containing amino acid in proteins. l-Met is also an important antioxidant because its sulfur group. Moreover, l-Met due to the presence of reactive species such as –S, –NH_2_ and –OH, shows a potential for reduction of oxygenise species. Hence, the study investigates the potential of l-Met for reduction of GO and Ag^+^. The prepared RGO–Ag nanocomposites were used as a catalyst for the reduction of 4-nitrophenol [4-NP] to 4-aminophenol [4-AMP]. 4-Nitrophenol and its derivatives are an important by-product from the production of pesticides, herbicides, and synthetic dyes and causes for a serious damage to the central nervous system, liver, kidney and blood of animals and humans. Thus, an effective removal of 4-NP from contaminated wastewater is one of the most important environmental issues. Furthermore, the antibacterial activities of RGO–Ag nanocomposite were also investigated against *B. subtilis* and *E. coli*.

## 2. Experimental

### 2.1 Chemicals

Graphite flake with +100 mesh was purchased from Sigma Aldrich. Silver nitrate (AgNO_3_), hydrogen peroxide (30 wt%), sodium nitrate (98%), sulphuric acid (98 wt%), potassium permanganate (KMnO_4_), and hydrochloric acid (HCl) (Merck, India) were used to synthesis graphene oxide (GO). l-Methionine (Himmide, India) used as a reducing and surface functionalization of GO and Ag^+^. All other chemicals used were analytical graded. Solutions were prepared with ultrapure water (Milli-Q) throughout the experiment.

### 2.2 Synthesis of RGO–Ag

GO was synthesised using as it is shown in S1.† The synthesis of RGO–Ag nanocomposite was followed *in situ* reduction of AgNO_3_ using l-Met as a reducing agent and RGO as a platform to deposit Ag NPs. Typically, GO solution was prepared by weighing 0.1 g of GO in 100 mL distilled water followed by ultrasonication for 60 min. The prepared aqueous GO solution (1 mg mL^−1^) was kept under stirring condition. Then, a 100 mL of aqueous solutions of AgNO_3_ were added drop-wise to GO vial. The solutions were stirred continuously for 30 minutes, and then 20 mL (10 mg mL^−1^) of l-Met solution was added. After 15 minutes of stirring, the pH of the solution was adjusted to 10 using NH_3_ (aq) and heated at 80 °C for 10 hours. The brown colour of the solution was turned into black upon the reaction time progressed. Then the black precipitate was centrifuged and washed with Mill-Q water several times and dried under vacuum at room temperature.

### 2.3 Characterization

The UV-Visible (UV-Vis) absorption spectra were recorded using a Shimadzu 2450 – SHIMADZU spectrometer. Fourier transform-infrared (FTIR) spectra were recorded over the range of 400–4000 cm^−1^ using a SHIMADZU-IR PRESTIGE-2 Spectrometer. Powder samples were mixed thoroughly with KBr and pressed into thin pellets. X-ray diffraction (XRD) patterns were recorded by PANalytical X'pert pro diffractometer at 0.02 degree per s scan rate using Cu-K_α1_ radiation (1.5406 A0, 45 kV, 40 mA). Transmission electron microscopy images were obtained (TEM model FEI TECNAI G2 S-Twin) at an accelerating voltage of 120 and 200 kV. The morphologies of the samples were characterized using field emission scanning electron microscopy (FESEM, Zeiss Ultra-60) equipped with X-ray energy dispersive spectroscopy (EDS).

### 2.4 Catalytic reduction of 4-nitrophenol to 4-aminophenol

The catalytic activity of the synthesized RGO–Ag nanocomposites was investigated by performing a model reduction reaction of 4-nitrophenol (4-NP) to 4-aminophenol (4-AMP) in the presence of sodium borohydride (NaBH_4_). Herein, the catalytic reduction of 4-NP by RGO–Ag nanocomposite in the presence of NaBH_4_ at different concentrations of 4-NP and different amount of RGO–Ag nanocomposite was investigated. Typically, in a quartz cuvette, 1 × 10^−3^ M of 4-NP (0.1 to 0.5 mL) was mixed with equal volume of freshly prepared 10–2 M NaBH_4_. To this mixture, 1 mg L^−1^ of RGO–Ag (0.05 mL) nanocomposite was added. Finally, Milli-Q water was added until 3 mL of a total volume of the solution was obtained. The reaction was monitored using UV-Vis spectrophotometer by observing the change in intensity of 4-nitrophenolate anion absorbance at 400 nm.

## 3. Result and discussion

### 3.1 Synthesis analysis

**Fig. 1:**
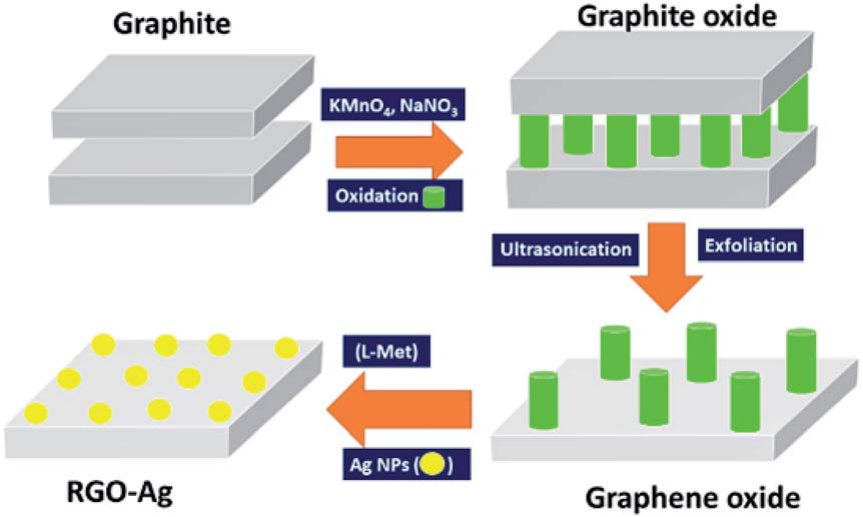
The schematic diagram of synthesis of RGO–Ag nanocomposite in the presence of l-Met.

The colloidal dispersion of graphene oxide (GO) solution synthesized using modified Hammer's methods predominantly exists negatively charged due to the presence of carboxylate groups,^35^ and as the same time silver salts in aqueous solution exist in cationic form (Ag_aq_^+^) is positively charged. When a colloidal solution of GO is added to Ag_aq_^+^ solutions, an electrostatic attraction leads to the self-assembly of cationic Ag_aq_^+^ colloids on the anionic GO surface, and resultant composites precipitated from solution. Reduction of Ag^+^ is the key step in the synthesis of the nanocomposite. Herein, instead of using noxious reducing agents, such as hydrazine, in this work l-Met was used for reduction of Ag^+^. l-Met is an essential sulphurous α-amino acid and not toxic to the environment. Furthermore, because of its enriched in chemistry (–S–, –NH_2_, and COO^−^), l-Met is effective for the reduction of both Ag^+^ and GO. l-Met in alkali media (above pI of l-Met = 5.74) found to be COO^−^ and NH_2_ forms and are responsible for the reduction of Ag^+^ and GO. In alkali media, GO is partially reduced graphene oxide (RGO) using l-Met *via* elimination of oxygen functional groups of GO. In addition, the intercalation of Ag NPs and l-Met between RGO layer, preventing the restacking of RGO and thus aggregation of RGO was arrested with high colloidal stability. The resultant RGO–Ag nanocomposite was easily filtered out through simple centrifugation and washed with distilled water. The schematic representation of synthesis of RGO–Ag nanocomposites in the presence of l-Met is presented in synthesis Fig. 1.

### 3.2 Characterizations analysis

**Fig. 2:**
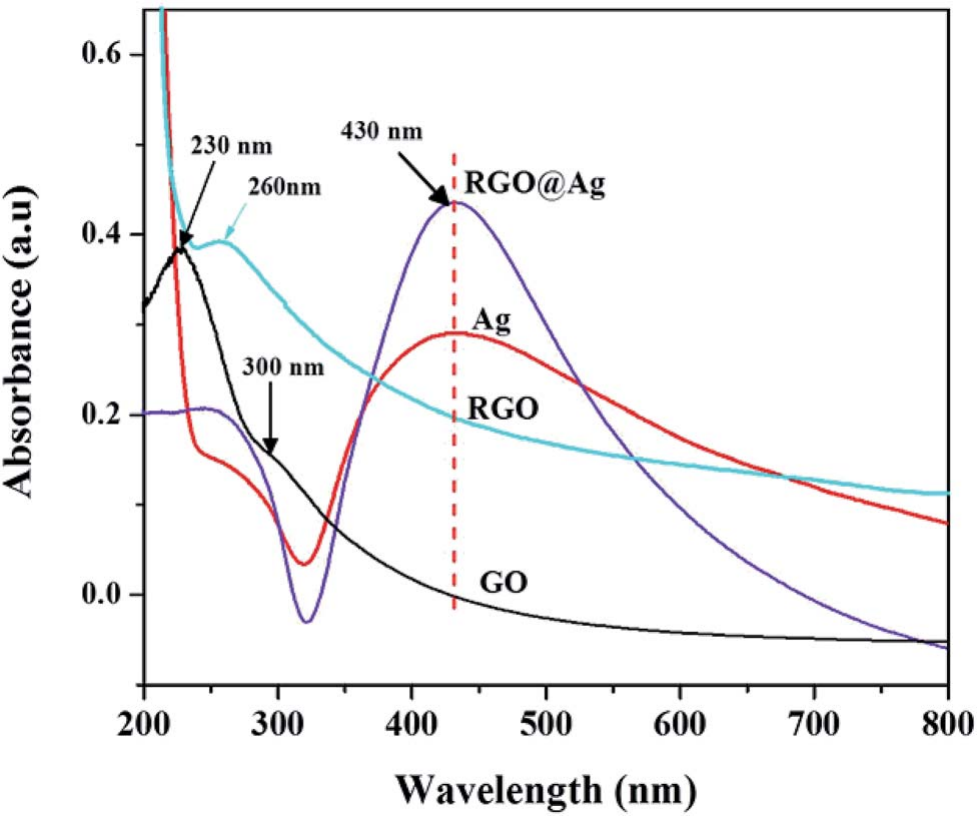
The UV-Vis spectra of GO, l-Met reduced GO (RGO–l-Met), l-Met reduced Ag NPs and RGO–Ag nanocomposites.

The absorption spectra of GO, l-Met–RGO, Ag NPs, and RGO–Ag nanocomposites are shown in Fig. 2. The characteristic absorption peaks at 230 nm and 300 nm are due to the π–π* transition of aromatic C–C and n–π* transition of C=O of GO. When GO is treated with l-Met, all the characteristic absorption peaks due to GO (230 nm and 300 nm) were absent and a new peak appeared at 260 nm, which confirms the successful reduction of GO to RGO by l-Met without any external reducing agent. The formation of new peak at 260 nm suggested that the restoration of the electronic conjugation of the graphene sheets occurs after treatment by l-Met. A similar redshift was observed for the reduction of GO using l-cysteine^36^ and l-ascorbic acid.^37^ As it is shown in Fig. 2, when l-Met reacted to Ag^+^ results in the formation of a peak at 431 nm, which is ascribed to the surface plasmon resonance (SPR) of Ag NPs.^38,39^ After the addition of l-Met into Ag^+^–GO solution, there was observed a new absorbance peak centred at 430 nm, which is assigned to the SPR peak of Ag colloids, indicating the formation of Ag nanoparticles.^38,40^

**Fig. 3:**
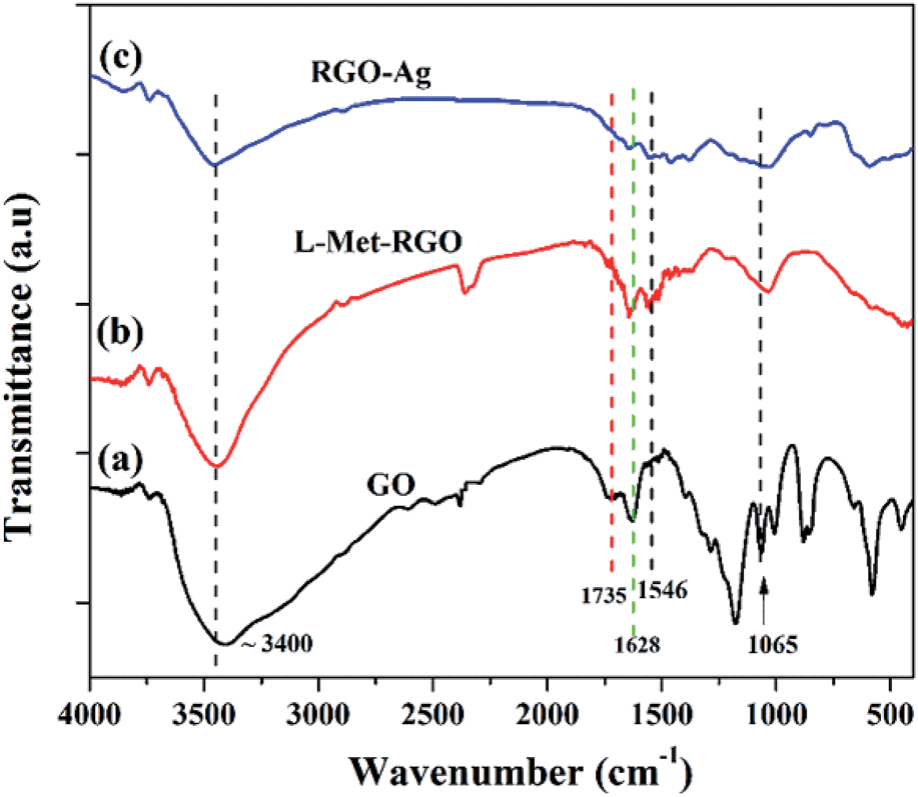
FTIR spectra of GO, l-Met reduced GO (l-Met–RGO), and RGO–Ag nanocomposite.

FTIR was used to evaluated the presence of functional groups present in GO, RGO, and RGO–Ag nanocomposite as it is shown in Fig. 3. The FTIR spectrum of GO shows the characteristic peaks at ∼3400 cm^−1^ (*ν*_O–H_), ∼1735 cm^−1^ (*ν*_C=O_), 1628 (*ν*_C–C_) and 1065 cm^−1^ (*ν*_C–O_).^41,42^ The formation of very weak intense peaks at 1735 cm^−1^ and 1065 cm^−1^ for l-Met reduced GO when compared to GO confirms the reduction of GO to RGO by l-Met. Moreover, the peak at 1628 cm^−1^ ascribed to the aromatic C=C group still exists. It shows that reduction by l-Met is well retained the C=C frame of sp^2^ hybridized carbon. l-Met–RGO shows a new peak at 1546 cm^−1^ (Fig. S2†) due to the N–H bending vibration. This is attributed that l-Met is attached to RGO surface. In the case of RGO–Ag, the peaks due to carbonyl and ether groups show a further reduction in their intensity, this affirms that the simultaneous reduction of GO and the formation of Ag NPs by l-Met.

**Fig. 4:**
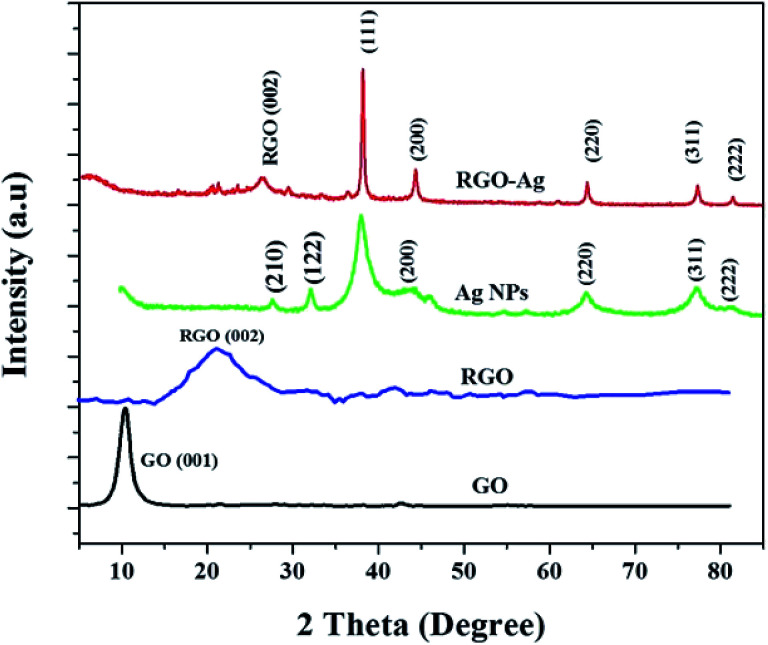
The powder XRD patterns of GO, l-Met reduced GO (l-Met–RGO), l-Met reduced Ag NPs and RGO–Ag nanocomposite.

XRD patterns are used to further study the crystalline structure of GO, l-Met–RGO, Ag NPs, and RGO–Ag nanocomposites. Fig. 4 shows the powder XRD pattern for GO, it has a very strong peak at 2*θ* = 10.5° which is ascribed to the (001) plane of GO, and its corresponding to an average interplanar distance found to be 0.78 nm. After reduction of GO to RGO using l-Met, a broad peak centred at 2*θ* = 24°, with a basal spacing of ∼0.34 nm instead of 0.78 nm for GO, which is due to the oxygen-containing functional groups are removed from GO.^33^ The XRD patterns of RGO–Ag nanocomposite has sharp diffraction patterns located at ∼38.3°, 44.2°, 64.4°, 77.4°, and 81.5° could be indexed as (111), (200), (220), (311) and (222) facets with a face-centered cubic (fcc) structure, which is well agreement with the standard Ag NPs JCPDS file (JCPDS 87-0597). l-Met–RGO–Ag shows a broad peak at 2*θ* = 26° which is indexed to the (002) facet of RGO. The reduction of GO to RGO by l-Met was further confirmed by the formation of an additional peak at ∼2*θ* = 25°, which is ascribed to (002) plane of RGO. The average crystallite size of the deposited Ag NPs on RGO was calculated from the (111) of RGO–Ag nanocomposite using Scherrer's equation found to be 23 nm.

**Fig. 5:**
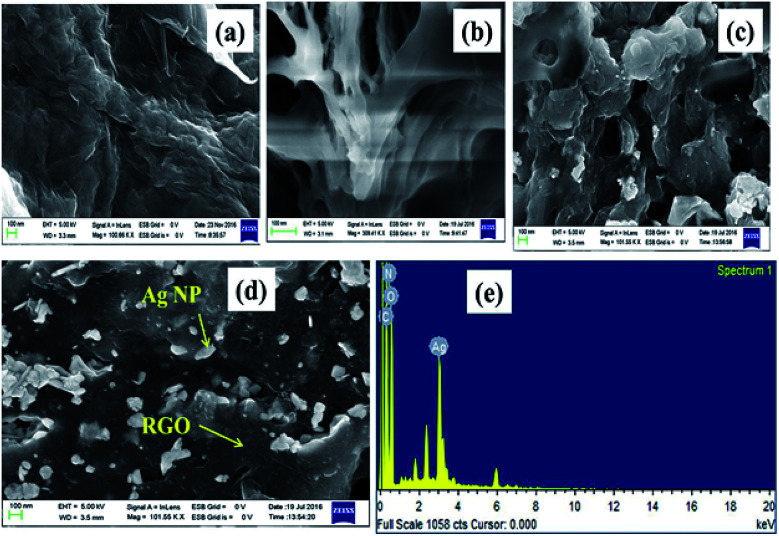
FESEM images of (a) GO, (b) l-Met reduced GO (l-Met–RGO), (c and d) RGO–Ag nanocomposite and (e) EDS spectrum of the RGO–Ag nanocomposite.

**Fig. 6:**
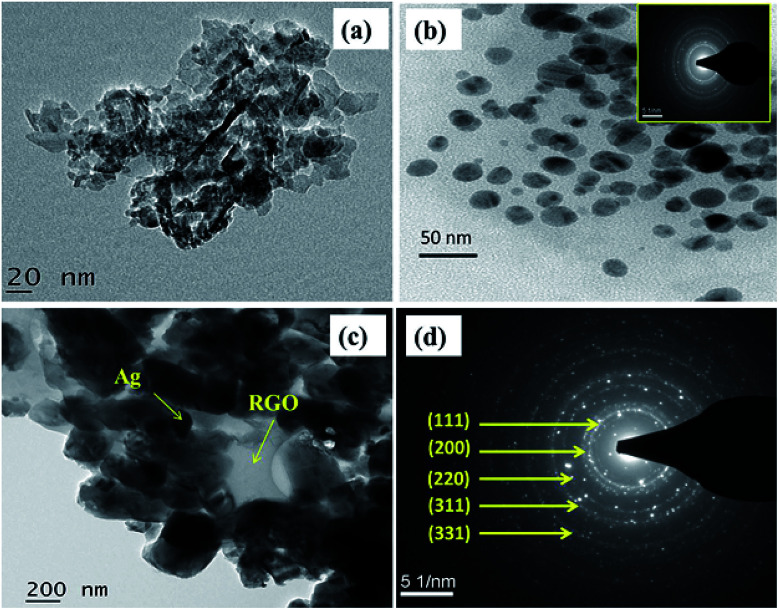
The representative TEM images of (a) l-Met reduced GO (RGO–l-Met), (b) l-Met reduced Ag NPs, (c) RGO–Ag nanocomposite and (d) SAED pattern of RGO–Ag.

The surface morphology of GO, l-Met–RGO and RGO–Ag were performed by FESEM and TEM. FESEM image reveals that (Fig. 5(a)), the stacked layer structure of GO. Unlike GO, l-Met capped RGO (Fig. 5(b)) shows exfoliated and highly wrinkled layers which are ascribed to the formation of a few layer RGO. Besides, the EDS spectrum of GO (Fig. S3†) and l-Met–RGO (Fig. S4†) show the elemental compositions. The oxygen content of l-Met–RGO is less than GO, which is ascribed that the partial reduction of GO using l-Met. Fig. 5(c and d), were the representative FESEM images of RGO–Ag nanocomposite, shows encapsulated Ag NPs by RGO layers dispersed over RGO layers. The EDS spectrum (Fig. 5(e)) reveals the presence of Ag, C, N, and O in the RGO–Ag nanocomposite. Furthermore, the morphology of the l-Met reduced GO, l-Met reduced Ag NPs and RGO–Ag nanocomposites were studied by TEM. As it can be seen in Fig. 6(a), RGO sheets are clearly exfoliated into a few layers. Fig. 6(b) shows the spherical shaped Ag NPs synthesized in the presence l-Met, which act as a reducing agent. As it is shown in Fig. S5,† the average particle size of pure Ag NPs was found to 13.17 nm. The inset image of Fig. 6(b) shows the SAED patterns of Ag NPs which in accordance with face-centered cubic (fcc) structure of Ag NPs. The TEM images of RGO–Ag nanocomposite as shown in Fig. 6(c), clearly indicates Ag NPs encapsulated by RGO sheets. The SAED patterns of RGO–Ag (Fig. 6(d)), which indicate the highly crystalline nature of Ag NPs and the diffraction patterns could be assigned to fcc structure of Ag NPs.

### 3.3 Catalytic reduction of 4-NP to 4-AMP

**Fig. 7:**
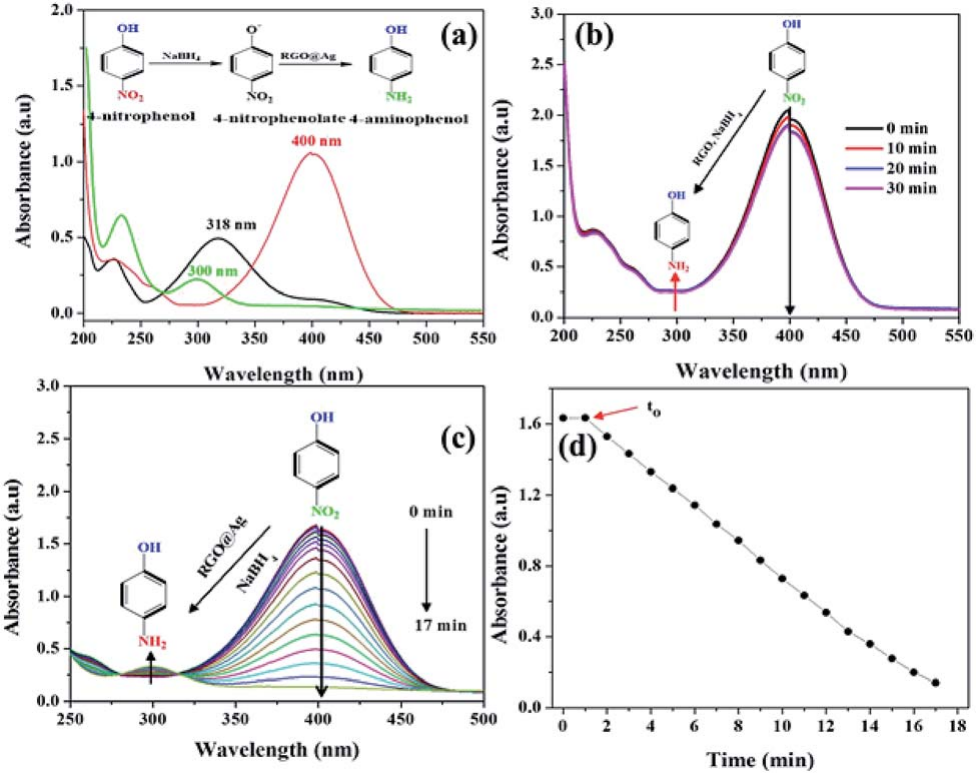
(a) The UV-Vis absorption spectra of 4-NP, 4-AMP and 4-nitrophenolate ion, (b) the catalytic reduction of 4-NP (1.67 × 10^−4^ molar) in the presence of NaBH_4_/RGO, (c) NaBH_4_/RGO–Ag nanocomposite, and (d) the correspondence real time absorbance of 4-NP at 400 nm in the presence of NaBH_4_/RGO–Ag.

The catalytic reduction efficiency of RGO–Ag nanocomposite towards the reduction reaction of 4-NP to 4-AMP was investigated by varying catalyst type, catalyst concentration and concentration of 4-NP. As it is shown in Fig. 7(a), the absorption peak of 4-NP (*λ*_max_ = 318 nm) was red-shifted to 400 nm in the presence of NaBH_4_, which is ascribed to the formation of the 4-nitrophenolate ion. As the reduction time progressed, the peak due to 4-nitrophenolate ion vanishes and concomitantly form a new peak around 300 nm, which is attributed to the formation of 4-AMP. The extent of 4-NP reduction was monitored by evaluating the decreasing in absorbance at 400 nm using UV-Vis spectroscopy. Fig. 7(b) shows the UV-Vis spectra of 4-NP in the presence of NaBH_4_/RGO mixture, there is no significant reduction of 4-NP absorbance at 400 nm within 30 minutes. Even though the reaction appears to be feasible in terms of the reduction potential of 4-NP (*E*^0^ for 4-NP/4-AP = −0.76 V) and NaBH_4_ (H_3_BO_3_/BH_4_^−^ = −1.33 V) *versus* normal hydrogen electrode (NHE), this process in not kinetically allowed. However, fascinatingly in the presence of NaBH_4_ and RGO–Ag nanocomposites (Fig. 7(c)), the yellow colour of 4-NP solution vanished quickly, monitored by the fast decrease in absorbance at 400 nm within a few minutes. At the same time, a new peak appeared at 300 nm, for 4-AMP and its absorbance increased with the reduction time. Hence, the presence of the Ag NPs is responsible for the reduction of 4-NP to 4-AMP. Fig. 7(d) shows the variation of the absorbance of 4-NP as a function of time. There is no distinguished change in the absorbance of 4-NP within the first 2 minutes, which is probably due to induction period (*t*_0_) of the catalyst. During this period, the RGO–Ag nanocomposites do not actively participate in the reduction reaction rather a structural reconstruction of the catalyst. Similar results have been reported in the literature.^43–45^ After the induction period, the reduction of 4-NP completes within 17 minutes. After 17 minutes, the reduction of 4-NP goes through a maximum, then no more absorbance of 4-NP was observed and where it indicates the end of the reduction reaction.

#### Kinetics of catalytic reduction of 4-NP

The kinetics of 4-NP reduction can be studied at different reaction parameters such as catalyst type, catalyst dose and 4-NP dose. The general rate law for the reduction of 4-NP to 4-AMP by NaBH_4_ is given by pseudo-first-order^46^ (eqn (1)),

1 −d[4-NP]/d*t* = *k*[4-NP]^*a*^[BH_4_^−^]^*b*^

*k*_app_ is the apparent pseudo-first-order rate constants is determined using equation (eqn (2)):

2 ln(*C*_0_/*C*) = *k*_app_*t*

where *C*_0_ and *C* are the initial and final concentrations of 4-NP. Therefore, a plot of ln(*C*_0_/*C*_*t*_) or ln(*A*_0_/*A*_*t*_) with respect to time gives a straight line whose slope is *k*_app_. The kinetic analysis was performed by a varying catalyst, the concentration of RGO–Ag and concentrations of 4-NP while keeping constant (BH_4_^−^).

#### Effect of catalyst

**Fig. 8:**
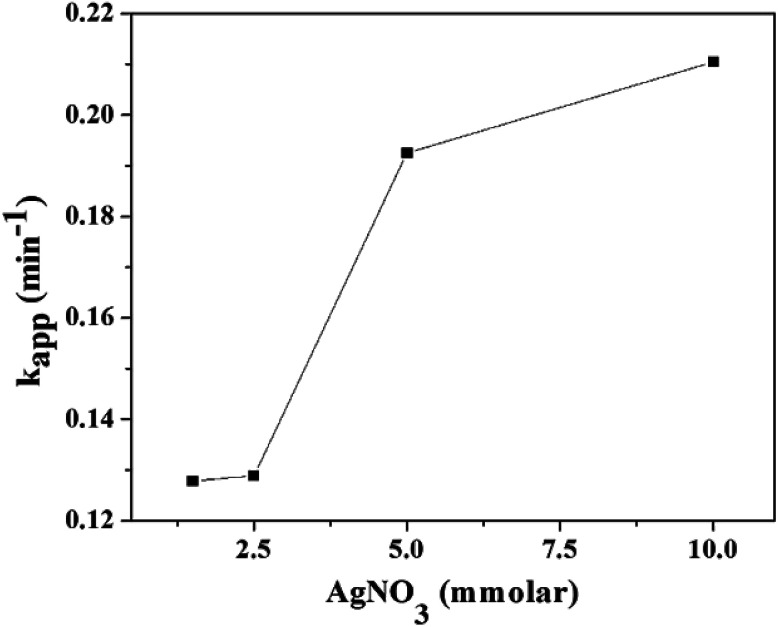
The pseudo-first order rate constant (*k*_app_) of the reduction of 4-NP to 4-AMP as function AgNO_3_ molar concentrations of RGO–Ag nanocomposites.

The RGO–Ag nanocomposite was synthesized by the varying molar concentration AgNO_3_ while the concentration of GO (1 mg mL^−1^) was kept constant (Table S1†). Fig. S6† shows the UV-Vis absorption spectra of the reduction of 4-NP to 4-AMP using different nanocomposites RGO–Ag in the presence of NaBH_4_. The linear plot of ln(*A*_*t*_/*A*_0_) as a function of reduction time (*t*) was obtained with *R*^2^ ≥ 0.97, which indicates the proposed mechanism (Scheme S1†) based on Langmuir–Hinshelwood^46^ sufficiently explained by pseudo-first order kinetics. The *k*_app_, as shown in Fig. 8 of 4-NP reduction increases while the molar concentration of AgNO_3_ increases.

#### Effect catalyst concentration

**Fig. 9:**
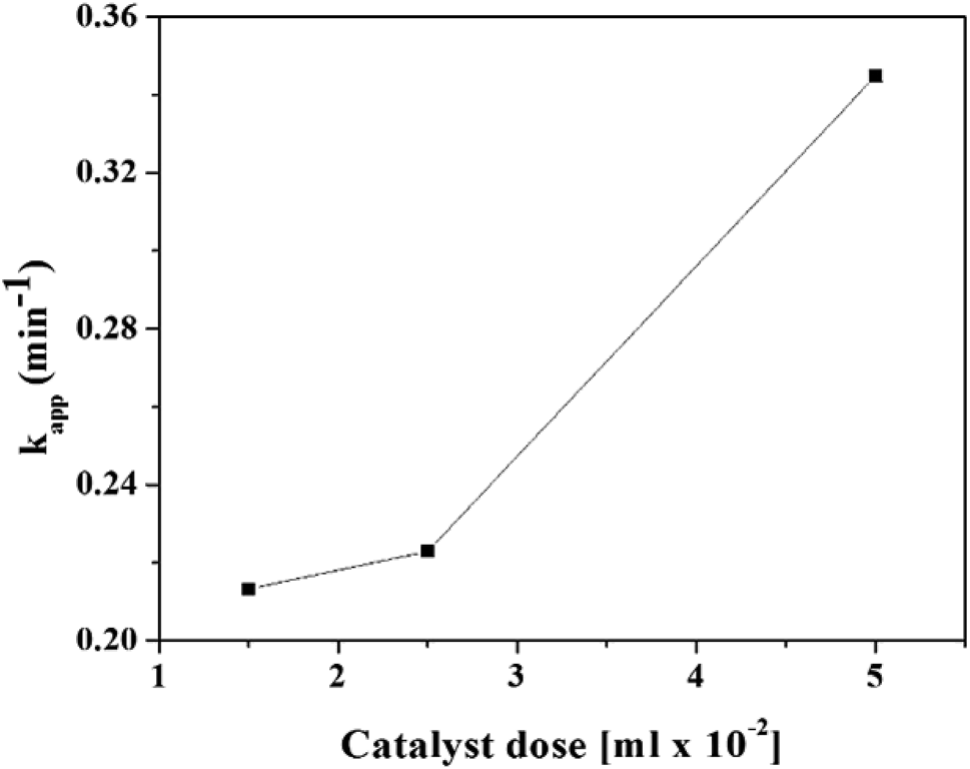
The pseudo-first order rate constant (*k*_app_) of the reduction of 4-NP to 4-AMP as a function of different concentrations of RGO–Ag nanocomposites.

The kinetics of reduction of 4-NP to 4-AMP was investigated as a function of RGO–Ag concentration. Fig. S7† shows the time-dependent UV-Vis absorption spectra of 4-NP reduction in the presence of various concentrations of RGO–Ag. The rate of reduction of 4-NP increases with the increases of RGO–Ag concentration and a linear plot of ln(*A*_*t*_/*A*_0_) *versus* reduction time was obtained. The increasing concentration is proportional to surface area, which enhance the rate reduction of 4-NP. Hence, as it is shown in Fig. 9, the *k*_app_ increases while increasing the concentration of RGO–Ag.

#### Effect of 4-NP concentration

**Fig. 10:**
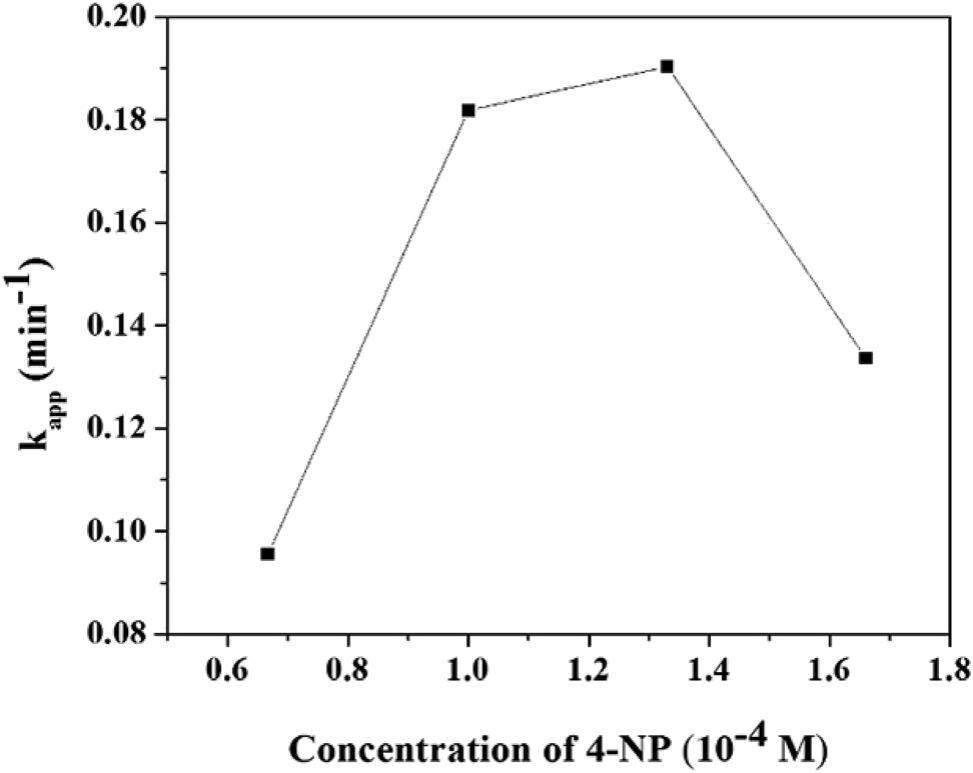
The pseudo-first order rate constant (*k*_app_) of the reduction of 4-NP to 4-AMP as a function of different concentrations of 4-NP.

The effect of 4-NP concentration on a change in the kinetic behaviour is recorded in Fig. S8.† Interestingly, the *k*_app_ increases with an increase in 4-NP from 0.067 × 10^−4^ to 1.33 × 10^−4^ and further increasing the concentration of 4-NP (1.33 × 10^−4^ to 1.67 × 10^−4^), it starts decreasing (as it is shown in Fig. 10). At very low concentrations of 4-NP, the surface is largely occupied by BH_4_^−^, which results in relatively lower values of the rate constant. With a further increase in the concentration of 4-NP the surface becomes increasingly occupied by more 4-NP molecules, causing an increase in the rate constant. The higher concentrations of 4-NP, however, interfere with the reaction with BH_4_^−^ due to higher binding constant for 4-NP compared to that of BH_4_^−^, resulting in a decrease in the rate constant.

#### Plausible reduction mechanism

**Fig. 11:**
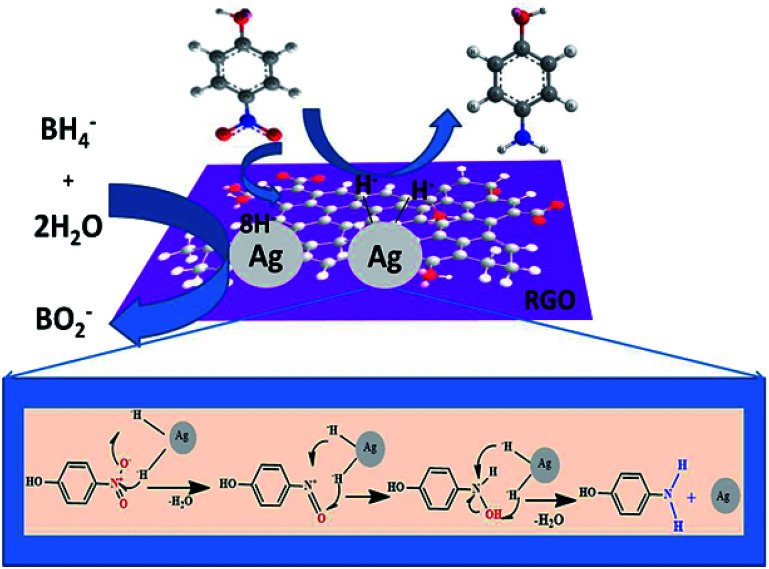
The schematic diagram of reduction of 4-NP to 4-AMP using RGO–Ag nanocomposite in the presence of NaBH_4_.

The reduction of 4-NP to 4-AMP using RGO–Ag nanocomposites in the presence of NaBH_4_ was performed *via* Langmuir–Hinshelwood (L–M) mechanism,^46^ assuming both the reactants adsorbed on the surface of RGO–Ag nanocomposite. As shown Scheme S1(i and ii),† 4-NP and BH_4_^−^, both substrates adsorbed on the surface of RGO–Ag nanocomposite. After getting interaction on the surface of the catalyst, undergo multiple steps for the reduction of 4-NP to 4-AMP by RGO–Ag nanocomposite in the presence of NaBH_4_. The adsorbed BH_4_^−^ reacts with water on the surface of a catalyst producing hydrogen as a reducing source and a reduction of adsorbed 4-NP and H takes place (iii) and forming 4-AMP on the surface of the RGO–Ag nanocomposite (iv), as it is shown Fig. 11. Finally, desorption of 4-AMP from the surface of the RGO–Ag nanocomposites (v) is the rate-determining step in the reaction proposed for the catalytic mechanism of RGO–Ag nanocomposite. The well-fitted of ln(*A*_*t*_/*A*_0_) *vs.* time (min) of kinetic analysis ascribes the L–M mechanism sufficiently to explain the kinetic of reduction of 4-NP to 4-AMP using RGO–Ag nanocomposite in the presence of NaBH_4_.

### 3.4 Antibacterial activity of RGO–Ag nanocomposites

**Fig. 12:**
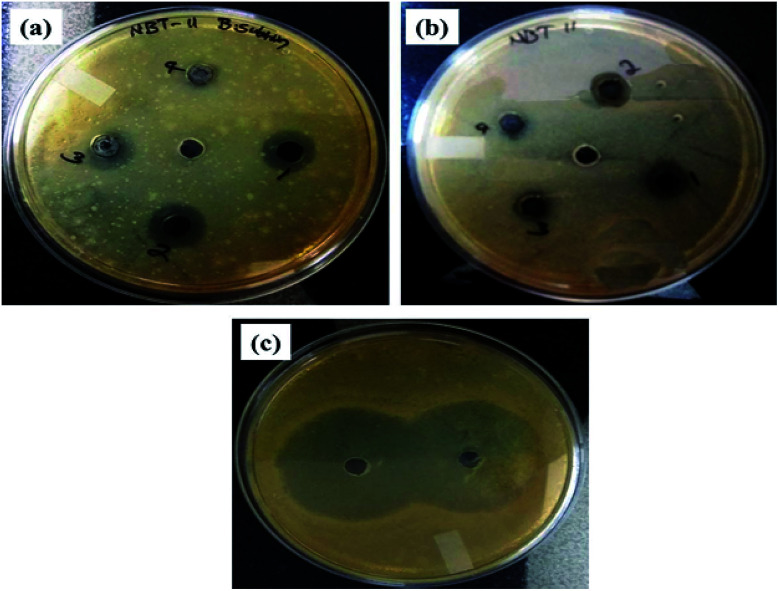
The images of antibacterial assay of RGO–Ag nanocomposite (a) *B. subtilis*, (b) *E. coli*, and (c) the antibacterial activity of the reference positive control.

**Fig. 13:**
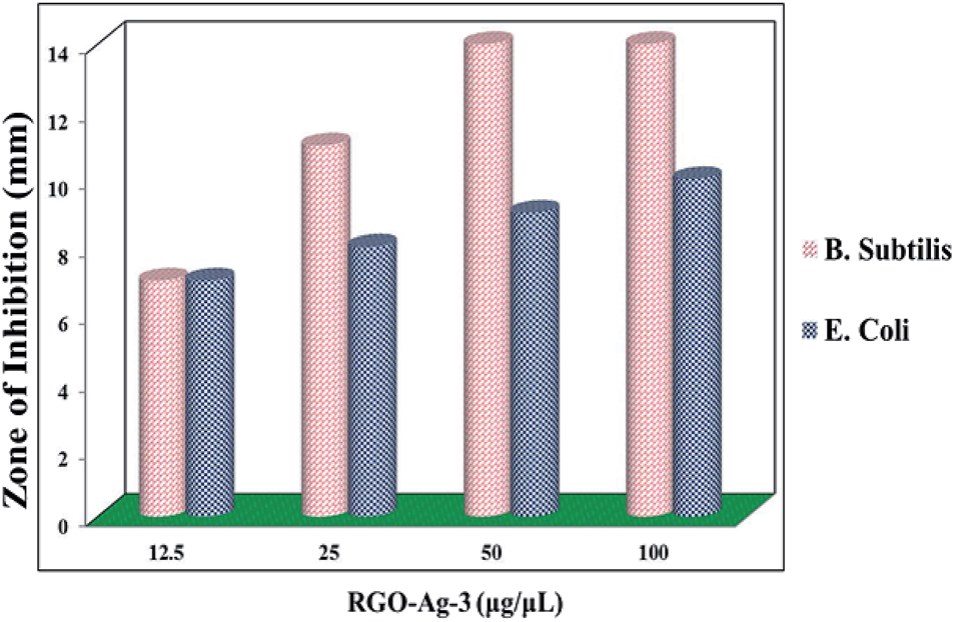
The antibacterial activity of different concentration of RGO–Ag nanocomposites towards *B. subtilis* and *E. coli*.

Assay of antibacterial activity of RGO–Ag nanocomposites was investigated against representative Gram-positive (*Bacillus subtilis*) and Gram-negative bacteria (*Escherichia coli*). Fig. 12 shows the inhibition zone for RGO–Ag nanocomposites for both bacteria. In the negative control, no zone of inhibition was found and in positive controls, a large zone of inhibition was found in both bacteria. Fig. 13 shows RGO–Ag nanocomposite exhibited a maximum zone of inhibition against *Bacillus subtilis* than against *Escherichia coli* and it seems that *Bacillus subtilis* more sensitive than *Escherichia coli*. Due to the strong bioactivity of these RGO–Ag nanocomposites, this work can be a basis for further valuable studies in the development of efficient antibiotics against the infections.

## 4. Conclusions

In conclusion, RGO–Ag nanocomposites were synthesized *via in situ* decoration of RGO by Ag NPs in the presence of l-Met as a reducing and stabilizing agent. The reduction of Ag^+^ to Ag NPs and GO to RGO was carried out by l-Met as a reducing agent. The formation of Ag NPs and RGO by l-Met was confirmed by UV-Vis, FTIR and EDS techniques. The phase purity and crystal structure of as synthesized RGO–Ag nanocomposites were confirmed by powder XRD. FESEM and TEM images were shown the encapsulation of Ag NPs by RGO sheet. The excellent catalytic reduction efficiency of RGO–Ag nanocomposites was investigated by the reduction reaction of 4-NP to 4-AMP as a model. Besides, RGO–Ag nanocomposites have shown a potential antibacterial activity against *B. subtilis* and *E. coli*.

## Conflicts of interest

There are no conflicts to declare.

## Supplementary Material

RA-009-C9RA08536J-s001
