# Supplementary material for: Green syntheses of silver nanoparticle decorated reduced graphene oxide using l-methionine as a reducing and stabilizing agent for enhanced catalytic hydrogenation of 4-nitrophenol and antibacterial activity
Source: RSC Adv. 2019 Nov 28;9(67):39264–71. doi: 10.1039/c9ra08536j (PMC9076085; doi:10.1039/c9ra08536j)
Supplement: RA-009-C9RA08536J-s001 [file RA-009-C9RA08536J-s001.pdf]

Electronic Supplementary Material (ESI) for RSC Advances.
This journal is © The Royal Society of Chemistry 2019

Green syntheses of silver nanoparticles decorated reduced graphene oxide using L-Methionine as a reducing and stabilizing agent for enhanced catalytic hydrogenation of 4-nitrophenol and antibacterial activity

Neway Belachew[a*], Desta Shumuye Meshesha[b], and Keloth Basavaiah[c]

[a] Department of Chemistry, Debre Birhan University, Debre Berhan, Ethiopia

[b] Department of Chemistry, University of Gondar, Gondar, Ethiopia

[c] Department of Inorganic & Analytical Chemistry, Andhra University, Vishakapatnam-530003

S1. Synthesis of GO

GO was synthesized based on the modified Hummer's method [1]. Particularly, three grams of graphite flake was first treated with acetone under the water bath sonicater for 30 minutes prior to oxidation. The modified graphite residue was dried at 50 ^{0}C for two hours. Then sonicated graphite (3 g) and $NaNO_3$ (1.5 g) were suspended in 69 mL concentrated sulfuric acid (18.4 M) in an ice bath (below 5 ^{0}C) in a 500 mL round bottom flask under gentle magnetic stirring for 15 min. Then 9 g of $KMnO_4$ was added continuously pinch by pinch in the above reaction mixture by keeping the temperature of the solution did not exceed than 20 ^{0}C. Then remove ice bath and the solution was stirred at 35 ^{0}C under a reflux condenser for 3 hours. After that, 130 mL of distilled water was added and stirring continued for an additional one hours. Excess unreacted $KMnO_4$ was removed by the addition of 15 mL of 30% hydrogen peroxide. The colour of the reaction mixture was turned from black (dark) to yellow, which indicated the complete removal

$KMnO_4$ from the reaction mixture. The as prepared GO carefully centrifuged and washed with deionized water at least three times and dried overnight using oven at 50 ^{0}C.

S2 Investigation of Antibacterial activity of RGO-Ag nanocomposites

The antibacterial activity of RGO-Ag nanocomposites were investigated against gram-negative (*Escherichia coli*) and gram positive (*Bacillus subtilis*) bacteria.

S2.1 Media preparation

The Mueller Hinton agar medium was used for bacteria development due to its acceptable reproducibility and satisfactory growth of most pathogens [12]. Typically, the agar medium was prepared by dissolving synthetic Mueller-Hinton agar powder in distilled water. The medium was adjusted to pH 7 with 1N NaOH and made up to 1L. The medium and Petri plates (100 mm x 15 mm) were autoclaved at 121 ^{0}C, 15 lbs for 20 minutes. The autoclaved medium was allowed to cool to 45 ^{0}C and transferred into Petri plates (20 ml/plate) under disinfected conditions of laminar air-flow.

S2.2 Agar-well diffusion testing

The standardized cultures of test bacteria were first evenly spread onto the surface of Mueller-Hinton Agar plates using sterile cotton swabs. Five wells (6 mm diameter) were made in each plate with a sterile cork borer. Fifty microliters of the nanocomposites were prepared in 4 different concentration ranges that are 100 μg/μL, 50 μg/μL, 25 μg/μL and 12.5 μg/μL and added in well No.1, 2, 3 and 4 each plate respectively (As shown in **Fig. S.1**). In the remaining well, 50 μl of 5 % DMSO (as a negative control) was added. And in a separate plate, 50 μl of reference antibiotic solution (as a positive control) were added. Gentamicin (200 μg/mL) and vancomycin (1 μg/mL) were used as reference antibiotics. Diffusion of nanocomposites, antibiotics, and DMSO was allowed at room temperature for 1 hour. All of the plates were then covered with lids

and incubated at 37 ^{0}C for 24 hours. After incubation, plates were observed for the zone of bacterial growth inhibition. The size of inhibition zones was measured and antimicrobial activity of the compounds was expressed in terms of the average diameter of inhibition zone in millimeters. Those compounds that were unable to exhibit inhibition zone (inhibition zone diameter less than 7 mm) were considered non-active.

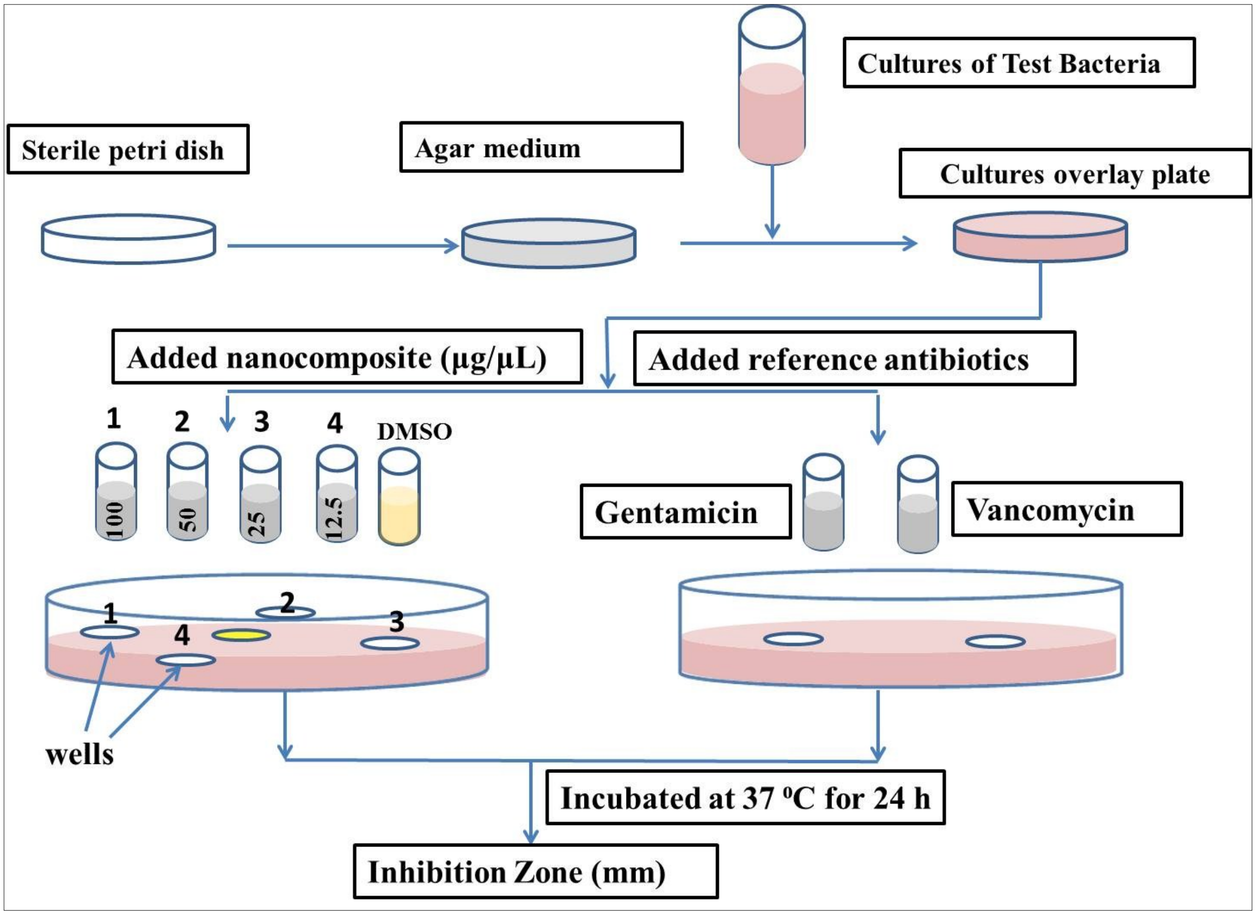

Fig. S1 The schematic diagram of agar-well diffusion method for testing antibacterial activity of RGO-Ag nanocomposites.

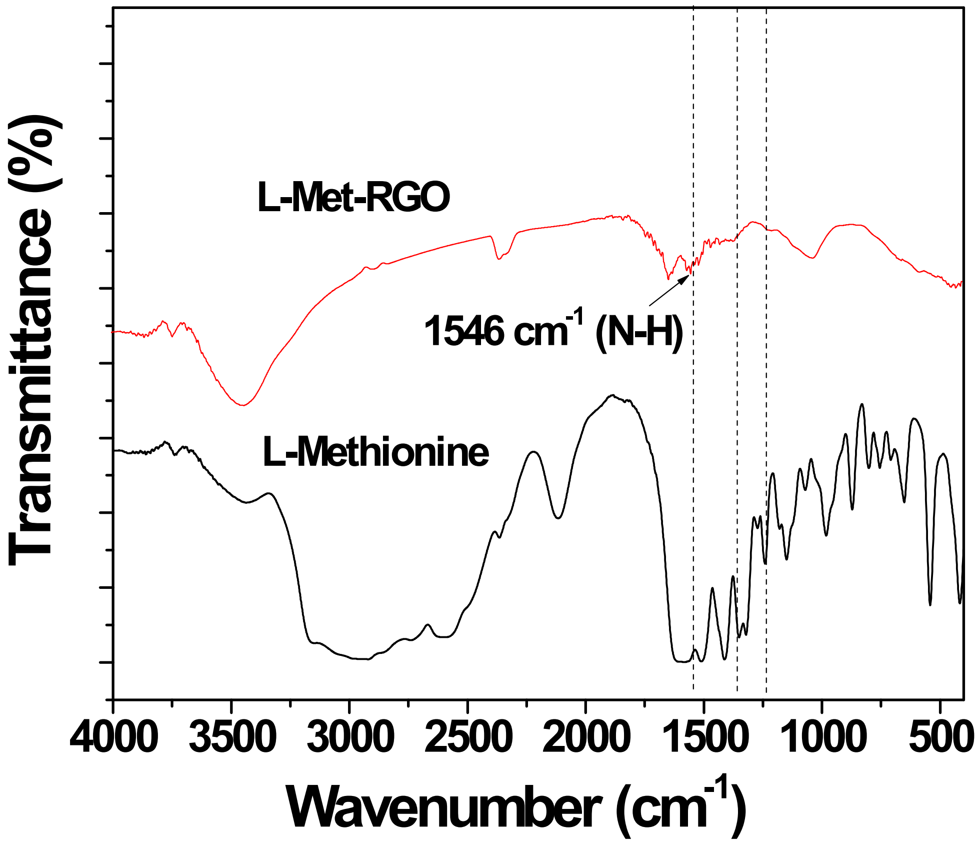

Fig.S2: The FTIR spectra of L-Methionine and L-Met reduced RGO. The vertical dot line shows the characteristics peaks of L-Methionine which are also observed in L-Met-RGO. This ascribed the L-Met is attached on RGO.

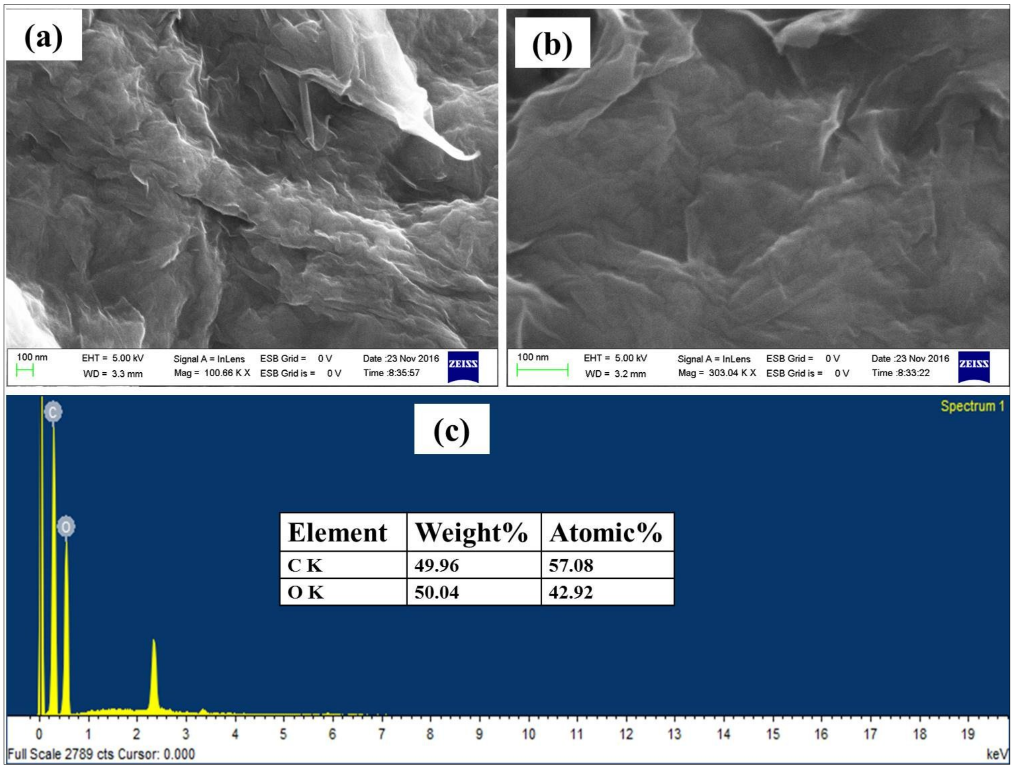

Fig. S3: (a) and (b) representative image and (c) the EDS graph of GO.

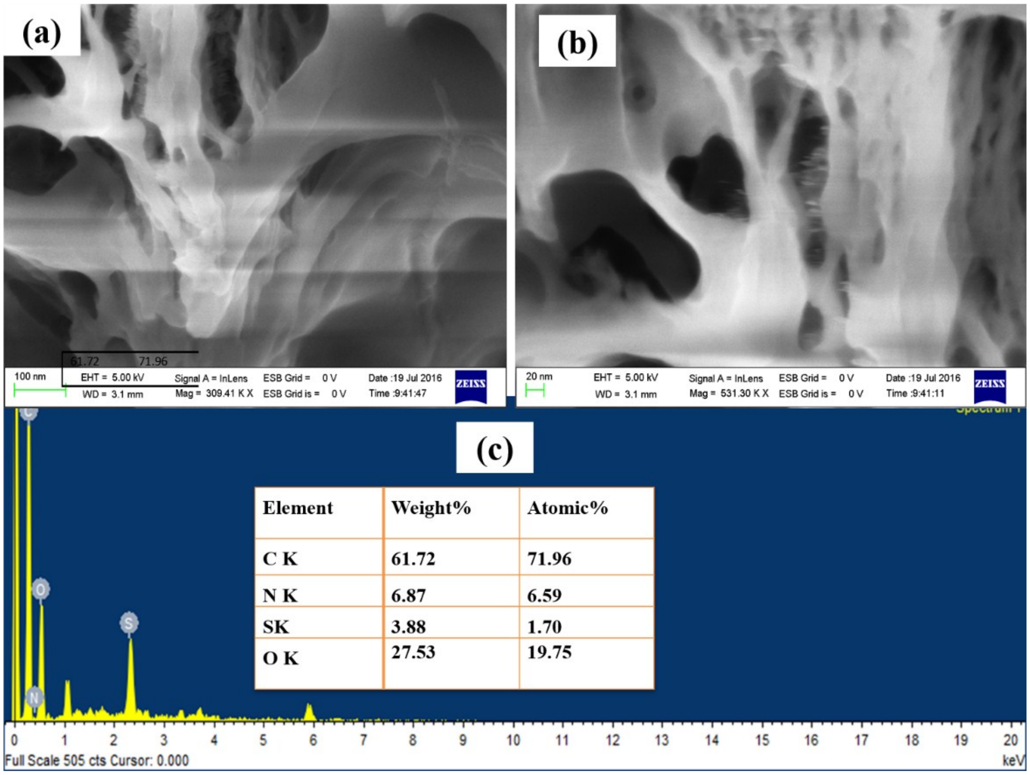

Fig.S4: (a) and (b) representative SEM images and (c) ESD spectrum of L-Met-RGO.

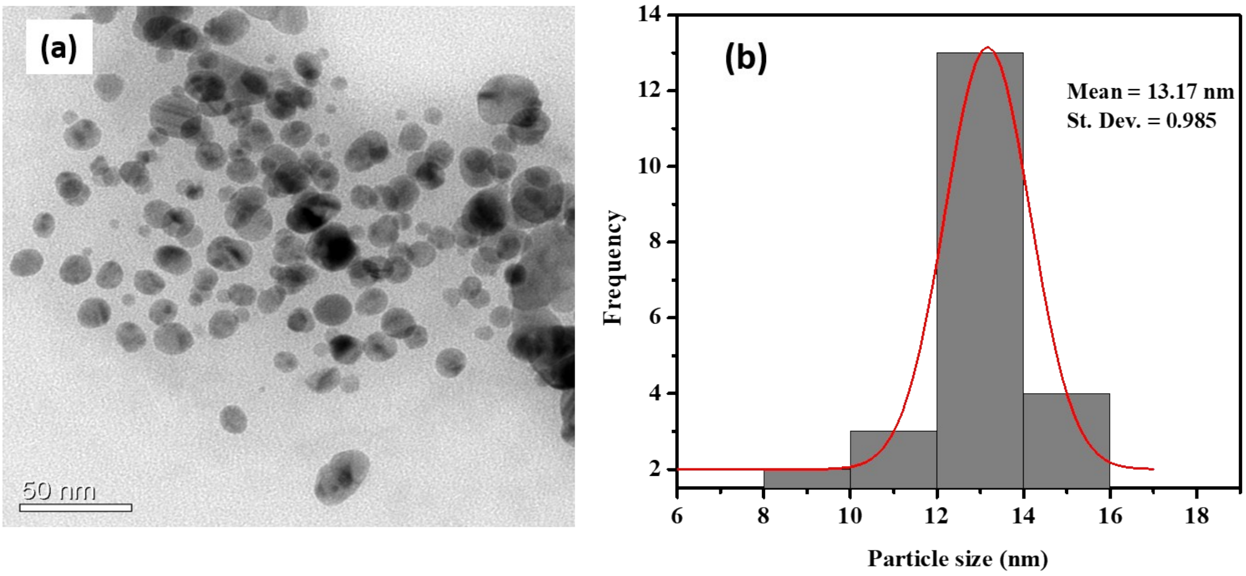

Fig.S5. (a) TEM image of Ag NPs and (b) the size distribution histogram of Ag NPs.

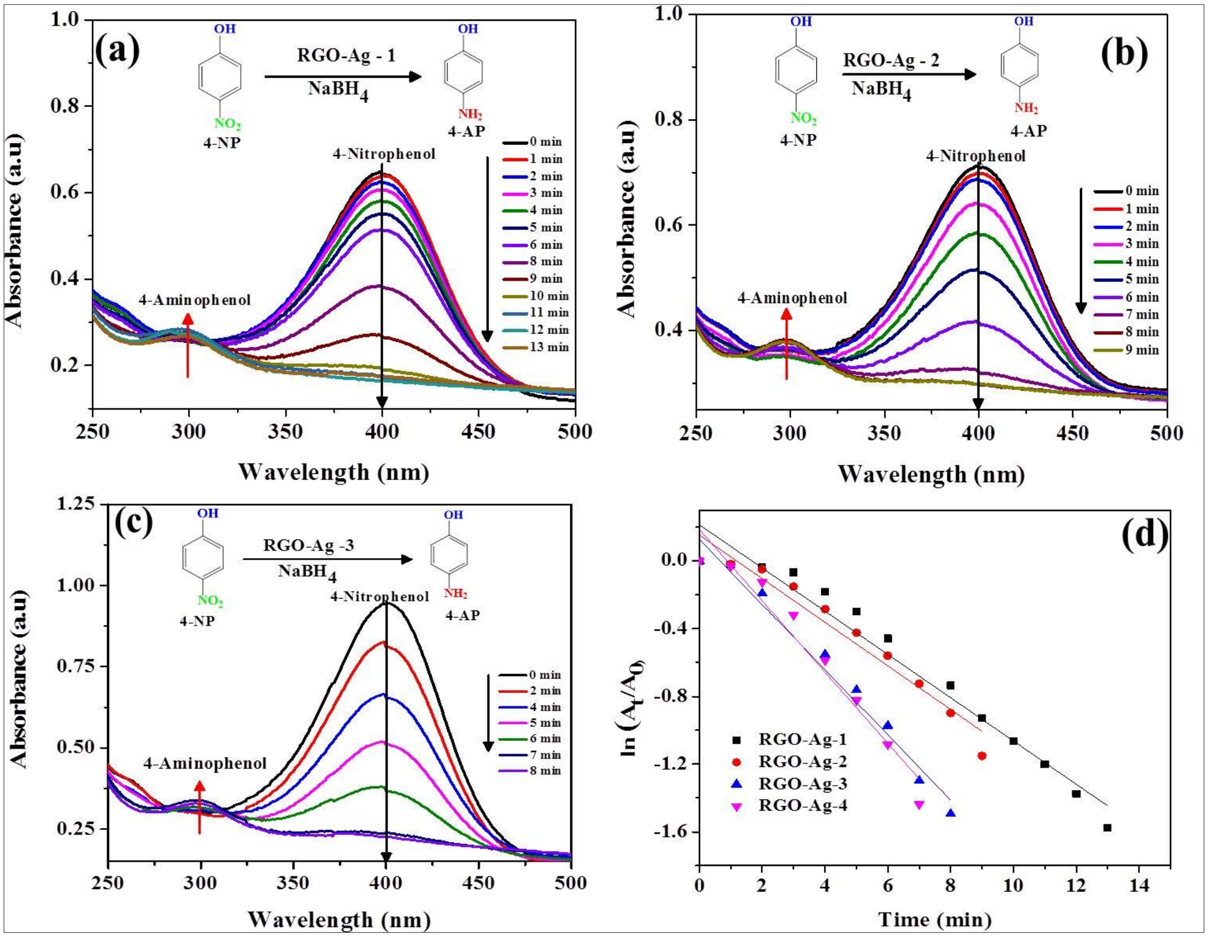

Fig. S6 (a-c) The time dependent UV-Vis absorption spectra, and (d) The linear plot of $\ln(A_t/A_0)$ as a function of time for the catalytic reduction of 4-NP (1.67×10^{-4} molar) to 4-AMP using RGO-Ag nanocomposites in the presence of $NaBH_4$.

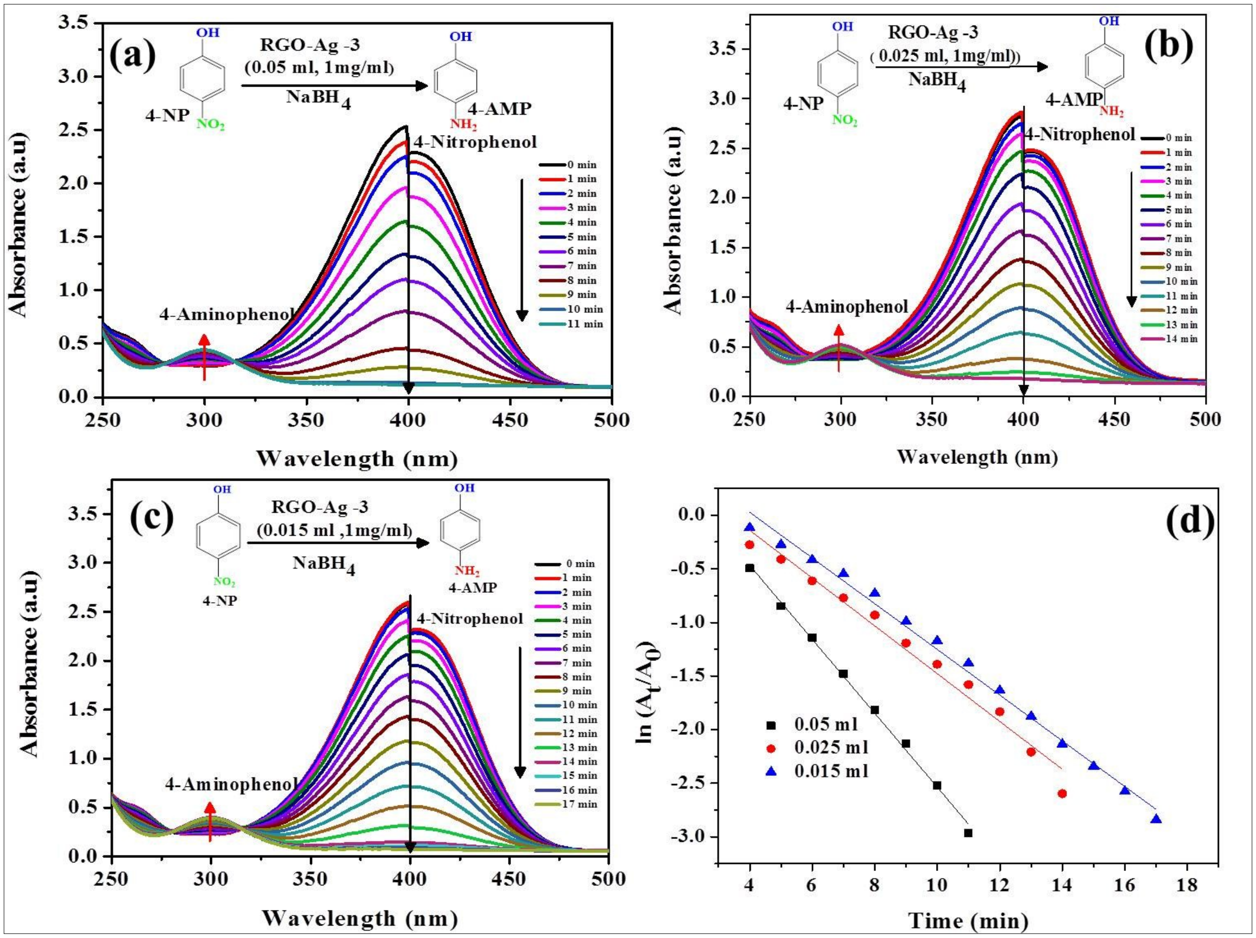

Fig. S7 (a-c) Time dependent UV-Vis absorption spectra, and (d) the linear plot of $\ln(A_t/A_0)$ as a function of time for the catalytic reduction of 4-NP (1.67×10^{-4} molar) to 4-AMP at different concentrations of RGOAg-3 nanocomposite in the presence of $NaBH_4$.

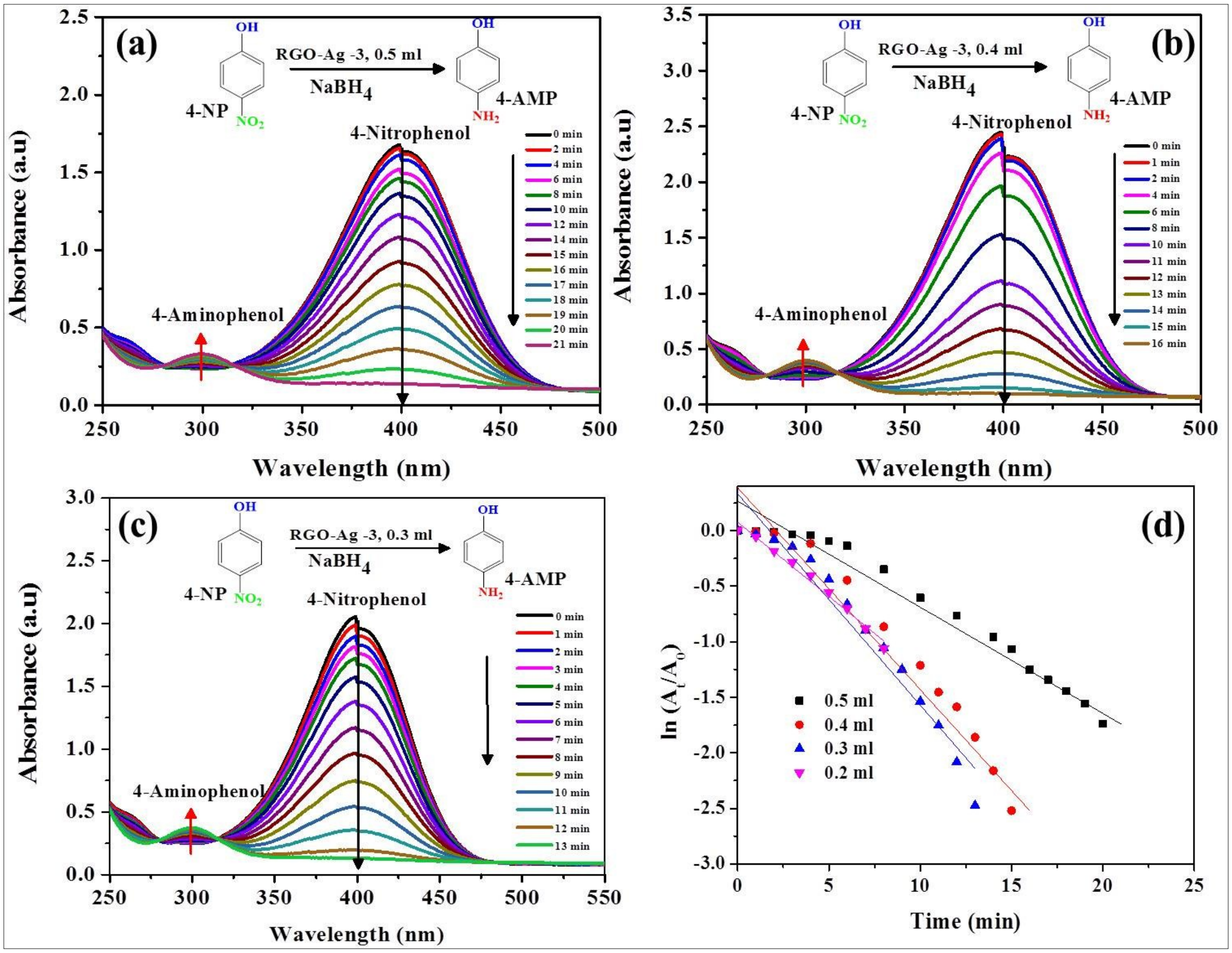

Fig. S8 (a-c) Time dependent UV-Vis absorption spectra, and (d) the linear plot of ln(A_t/A_0) as a function of time for varying concentration of 4-NP (0.67 x 10^{-4} to 1.67 x 10^{-4} molar) using RGO-Ag-3 nanocomposite in the presence of $NaBH_4$.

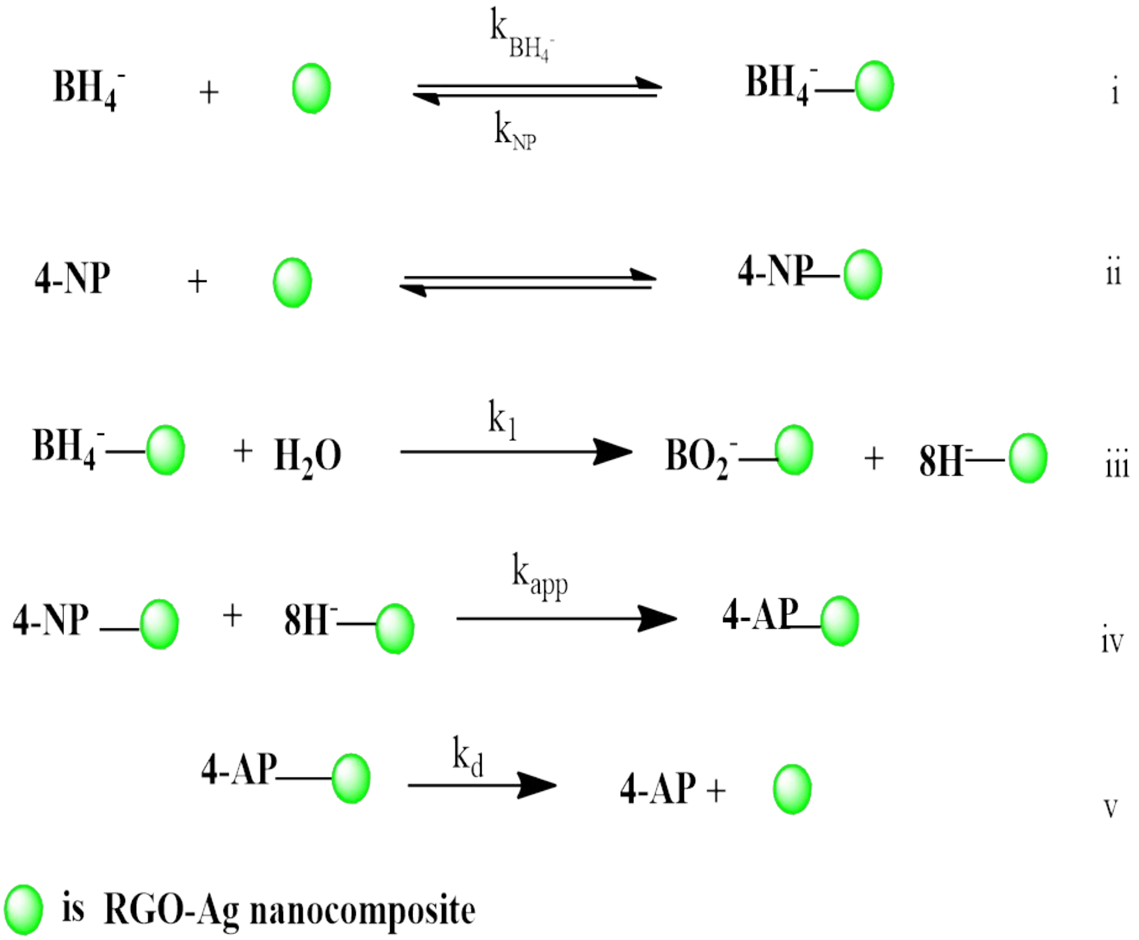

Scheme S.1 The Plausible reduction mechanism of 4-NP to 4-AMP using RGO-Ag/BH_4^-.

Table S1: **The synthesis condition and nomenclature of RGO-Ag nanocomposites.**

GO (g)	$AgNO_3$ solution		GO : Ag^+(mass ratio)
	mmolar	g	
**0.1**	1.5	0.0255	1:0.255
**0.1**	2.5	0.0425	1:0.425
**0.1**	5	0.085	1:0.85
**0.1**	10	0.17	1:1.7
